# Antiamoebic Activity of Imidazothiazole Derivatives against Opportunistic Pathogen *Acanthamoeba castellanii*

**DOI:** 10.3390/antibiotics11091183

**Published:** 2022-08-31

**Authors:** Noor Akbar, Mohammed I. El-Gamal, Balsam Qubais Saeed, Chang-Hyun Oh, Mohammed S. Abdel-Maksoud, Naveed Ahmed Khan, Ahmad M. Alharbi, Hasan Alfahemi, Ruqaiyyah Siddiqui

**Affiliations:** 1 College of Arts and Sciences, American University of Sharjah, University City, Sharjah 26666, United Arab Emirates; 2 Sharjah Institute for Medical Research, College of Medicine, University of Sharjah, Sharjah 27272, United Arab Emirates; 3 Department of Medicinal Chemistry, College of Pharmacy, University of Sharjah, Sharjah 27272, United Arab Emirates; 4 Department of Medicinal Chemistry, Faculty of Pharmacy, Mansoura University, Mansoura 35516, Egypt; 5 Department of Clinical Sciences, College of Medicine, University of Sharjah, Sharjah 27272, United Arab Emirates; 6 Center of Biomaterials, Korea Institute of Science and Technology (KIST School), Seongbuk-gu, Seoul 02792, Korea; 7 University of Science and Technology (UST), Yuseong-gu, Daejeon 34113, Korea; 8 Medicinal & Pharmaceutical Chemistry Department, Pharmaceutical and Drug Industries Research Division, National Research Centre (NRC), Dokki, Giza 12622, Egypt; 9 Department of Clinical Laboratory Sciences, College of Applied Medical Sciences, Taif University, Taif 21944, Saudi Arabia; 10 Department of Medical Microbiology, Faculty of Medicine, Al-Baha University, Al-Baha 65799, Saudi Arabia

**Keywords:** *Acanthamoeba castellanii*, amoebicidal, encystation, excystation, cytotoxicity, cytopathogenicity, imidazothiazole

## Abstract

We examined the antiamoebic effect of several imidazothiazole derivatives on *Acanthamoeba castellanii* of the T4 genotype. Trypan blue exclusion assays and haemocytometer counting were used to determine the reduction in *A. castellanii* trophozoite proliferation, in response to treatment with these compounds. To determine the effects of these compounds on host cells, lactate dehydrogenase assay was performed using HeLa cell lines. Amoebicidal assays revealed that the tested compounds at concentrations of 50 µM significantly inhibited amoebae trophozoites compared to controls. Compounds **1m** and **1zb** showed the highest amoebicidal effects eradicating 70% and 67% of *A. castellanii*, respectively. The compounds blocked both the encystation and excystation process in *A. castellanii*. Compounds **1m** and **1zb** blocked 61% and 55%, respectively, of amoeba binding to human cells. Moreover, the compounds showed minimal cytotoxic effects against host cells and considerably reduced amoeba-mediated host cell death. Overall, our study revealed that compounds **1m** and **1zb** have excellent antiamoebic potential, and should be considered in the development of curative antiamoebic medications in future studies. Further work is critical to determine the translational value of these findings.

## 1. Introduction

*Acanthamoeba castellanii* belongs to the genotype T4 and causes a blinding infection of the eye known as *Acanthamoeba* keratitis (AK) [1]. AK is a devastating eye infection that promotes corneal ulcers and can lead to permanent loss of vision or blindness [2,3,4]. The use of contact lenses as well as inadequate contact lens cleanliness are risk factors for *Acanthamoeba* keratitis [5]. Furthermore, *Acanthamoeba* can also cause a fatal infection of the central nervous system (CNS) known as granulomatous amoebic encephalitis (GAE). *Acanthamoeba* is widespread and can be found naturally in air, soil, and water, and in swimming pools (that are not properly chlorinated), tap and shower water, and even hot tubs [4]. Amoebae exist in two alternate stages; the infective trophozoites and double-walled dormant hardy cysts [6]. 

In AK, the amoeba attacks the epithelium first then the stroma. perineuritis, ring infiltrates, and dendrite-like components can all be detected [7]. AK diagnosis is based primarily on culture or, more recently, polymerase chain reaction (PCR) [7]. Corneal in vivo confocal imaging can be used as a supplement in some circumstances, but very often, the diagnosis is based purely on the clinical manifestations. Patients frequently experience a sluggish recovery and many depressive episodes. The prognosis may be poor, necessitating hot transplanting and, in the worst-case scenario, surgical removal [7].

Antimicrobial topical treatments, including different combination of biguanides, neomycin and propamidine isethionate are being used to treat *Acanthamoeba* keratitis [2,8]. The duration of these therapies, however, makes the process difficult. Moreover, because the treatments are ineffective against the protozoan cyst stage, persistent infection is common in these instances [6,9,10]. This necessitates the development of novel drugs and treatment regimens that are effective against both *Acanthamoeba* trophozoites and cysts [2,8,11,12].

Previously, the antiproliferative activity of imidazothiazole derivatives was reported against melanoma and other cancer types [13,14,15,16,17]. Their mechanism of action is via the inhibition of rapidly accelerated fibrosarcoma (RAF) kinases including V600E-B-RAF and C-RAF. Furthermore, they showed ability to penetrate the cell membrane and inhibit the kinase inside melanoma cells. In this study, the antiamoebic activity of these compounds were investigated against *Acanthamoeba castellanii* based on their cell membrane-penetrant properties.

## 2. Materials and Methods 

### 2.1. Compounds

The target imidazothiazole derivative compounds were synthesized and characterized as reported previously [13,14,15,16,17].

### 2.2. Acanthamoeba castellanii Cultures

*A. castellanii* genotype T4 (ATCC 50492) trophozoites were used in this study. The amoebae were axenically cultivated at 30 °C in T-75 cm^3^ culture flask using a proteose-peptone yeast extract-glucose (PYG) broth culture. Upon confluency, amoeba cultured flask was placed on ice for 10 min followed by gently tapping to dislodge the adhered trophozoites. Next, culture was centrifuged at 3000× *g* for 5 min. The supernatant was discarded and the pellet was re-suspended in 1 mL of Roswell Park Memorial Institute (RPMI) media. Finally, amoebae were counted via haemocytometer microscopically and the desired initial inoculum (5 × 10^5^) was adjusted and used in different assays.

### 2.3. Amoebicidal Assays

Different molecules were tested against *A. castellanii* to examine their antiamoebic effects as previously reported [1,18]. Briefly, *A. castellanii* (5 × 10^5^) were incubated with 50 µM of the tested compounds for 24 h at 30 °C with a final assays volume of 0.5 mL in a 24-well plate. For controls, amoebae cultured in RPMI alone and with 0.25% sodium dodecyl sulfate (SDS) was taken as negative and positive control, respectively. Finally, each well was stained with Trypan blue (0.1%) and viable amoebae trophozoites were enumerated by haemocytometer. In some trails, these compounds were tested at different concentrations (12.5, 25, 50 and 100 µM) to determine their 50% inhibitory concentration (IC_50_) against *A. castellanii* [19].

### 2.4. Henrietta Lacks Cervical Adenocarcinoma (HeLa) Cell Lines Cultivation

HeLa cells were obtained from the American Type Culture Collection (ATCC CCL-2) and grown at 37 °C with 5% CO_2_ and 95% humidity in RPMI supplemented with 10% fetal bovine serum (FBS), 1% minimum essential medium amino acids, 1% Penicillin-Streptomycin (Pen-Strep) and 1% L-glutamine. The media was removed aseptically, and cells were detached enzymatically using 2 mL of trypsin EDTA. Next, culture containing dislodged cells was centrifuged at 2500× *g* for 5 min. The cell pellet was resuspended in the above-mentioned media, subsequently added into 96-well plates and utilized in different assays.

### 2.5. Adhesion Assays

Adhesion assays were performed to determine how the tested molecules affect the binding capability of amoebae to human cells [20,21]. Firstly, *A. castellanii* (5 ×10^5^) trophozoites were treated with 50 µM of the compounds in serum-free RPMI medium at 30 °C for 2 h. Next, pre-treated amoebae were centrifuged at 3000× *g* for 5 min and re-suspended the amoeba in 200 µL of RPMI medium. The total assay volume including pre-treated amoeba was transferred to a confluent HeLa cell monolayer cultured in 96-well plates. For control, amoebae trophozoites alone were added to HeLa cell monolayer. The plates were incubated for 60 min at 37 °C with 5% CO_2_ and 95% humidity and amoebae (unbound) were then counted by haemocytometer. Percent bound amoebae was calculated using the following formula:

% amoebae (bound) = 100 − amoebae (unbound)(1)

### 2.6. Encystation Assays

Encystation assays were performed to determine the effects of the tested compounds to prevent the differentiation of *A. castellanii* trophozoites into the cyst form and 50 µM of the tested compounds were utilized [21,22]. Briefly, *A. castellanii* (1 × 10^6^) trophozoites were incubated with these compounds with 16% filter-sterilized glucose (as encystation medium) at 30 °C 48–72 h. *A. castellanii* cultured alone in 16% glucose was taken as negative control. After this incubation, 0.1% sodium dodecyl sulfate (SDS) was added to each well and agitated the plate for 20 min. The SDS resistant cysts were enumerated using a haemocytometer and data was recoded. 

### 2.7. Excystation Assays

For excystation assays, *A. castellanii* trophozoites suspended in 3 mL phosphate buffered saline (PBS) was grown on non-nutrient bacteriological agar plates. The plates were incubated at 30 °C for 14 days and regularly observed for cysts formation [21,22]. Next, amoeba cysts were scrapped by cell scraper in PBS and culture containing cysts was centrifuged at 3000× *g* for 10 min. The cysts pellet was resuspended in RPMI and used in excystation assays. To inspect the excystation process, *A. castellanii* (1 × 10^5^) cysts were treated with 50 µM of the tested compounds using PYG medium (final volume 0.5 mL). For negative control, cysts were grown in PYG alone. The plates were incubated and routinely observed at 30 °C for 24–72 h. Finally, cysts transformed into amoebae trophozoites were counted and the data were recorded using a haemocytometer.

### 2.8. In Vitro Cytotoxicity Assays

In vitro cytotoxic effects of the tested molecules were determined using Lactate dehydrogenase (LDH) assays against human cells [8,23]. In brief, 50 µM concentration of the tested compounds were incubated with HeLa cells monolayer for 24 h at 37 °C with 5% CO_2_ in humidified conditions. After this incubation, 0.1% triton X-100 was added to the positive control wells and incubated the plate for 45 min at 37 °C. Then, an equal volume of cells supernatant containing liberated LDH enzyme was mixed with an equal volume of LDH kit reagents (Cytotoxicity Detection kit; Roche Diagnostics, Indianapolis, IN, USA). The positive and negative controls were cell monolayers treated with 0.1 percent Triton X-100 and in RPMI alone. The plate was subjected to multi-plate reader and absorbance was recorded at 490 nm. The percent cytotoxicity was calculated using given formula; % Cell cytotoxicity = sample value − negative control value/positive control value − negative control value × 100.

Additionally (3-(4,5-dimethylthiazol-2-yl)-2,5-diphenyltetrazolium bromide or MTT assays were performed to determine the 50% effective concentration (EC_50_) or 50% cytotoxic concentration (CC_50_) and maximum non-toxic dose (MNTD_90_) of the compounds with effective antiamoebic activity. These compounds were tested at different concentration (12.5 µM, 25 µM, 50 µM and 100 µM) to evaluate the CC_50_ and MNTD_90_ [23,24].

### 2.9. Amoeba-Mediated Host Cells Cytotoxicity

Cytopathogenicity assays were accomplished to determine the amoeba-mediated host cell death [21,22,25]. *A. castellanii* (5 × 10^5^) trophozoites were treated with 50 µM of the tested molecules at 30 °C for 2 h. Culture containing pre-treated amoebae was centrifuged at 3000× *g* for 5 min and pellet was re-suspended in 200 µL of serum free RPMI. The whole assay volume with pre-treated amoebae was transferred to HeLa cell monolayer cultured in a 96 well plates. Next, the plates were kept for overnight at 37 °C with 95% humidity and 5% CO_2_. Finally, amoeba-mediated host cell cytotoxicity was evaluated indirectly by measuring the amount of LDH enzyme released into cell media by damaged cells, as reported earlier [26,27].

### 2.10. Statistical Analysis

All statistical analysis were done using two-sample student *t*-test, two-tailed distribution. The data is presented as the mean standard error of several replicated studies. Graph Pad Prism version 8.0.2 was used for all the analyses and visualizations (GraphPad Software; San Diego, CA, USA). *p* ≤ 0.05 was used as the statistical significance level.

## 3. Results

### 3.1. Compounds Showed Significant Amoebicidal Activity against A. castellanii

**Figure 1:**
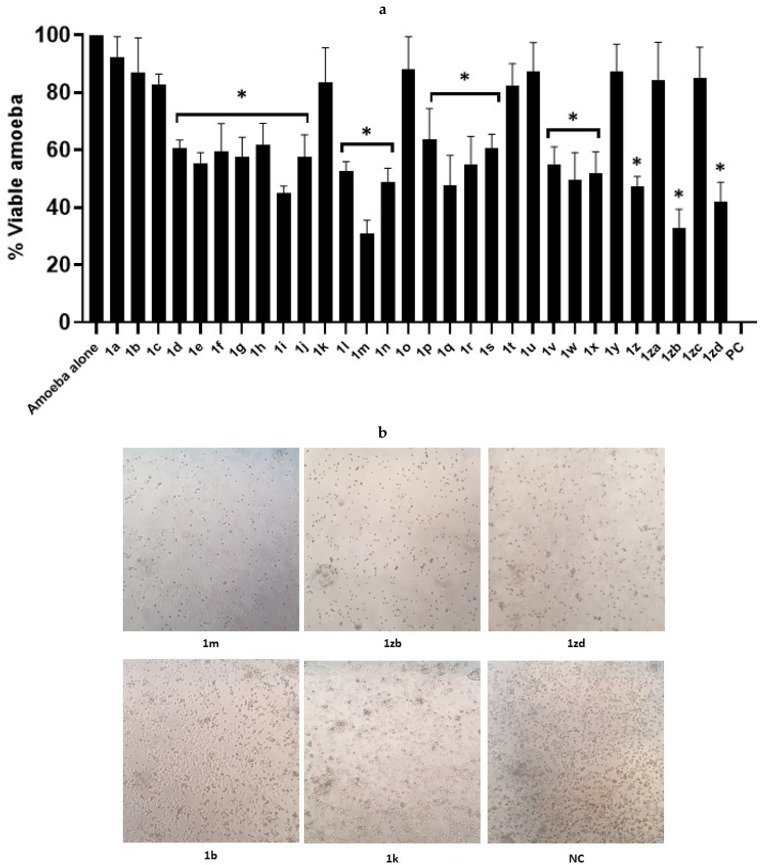
Compounds with notable amoebicidal activities against *A. castellanii*. Briefly, compounds were incubated at 50 µM concentration with *A. castellanii* (5 × 10^5^) at 30 °C for 24 h. Next, viable amoebae were enumerated by haemocytometer using inverted microscope. Amoebae cultured in RPMI alone was considered as negative control and with 0.25% SDS as positive control. (**a**) Amoebicidal effects of compounds and their analogues against *A. castellanii*. (**b**) Illustrative effects of compounds and their analogues on the amoeba viability. The NC represent the negative control (amoebae alone) while PC indicate the positive control. Images are shown at 100×. The data is expressed as the mean ± standard error. *p* values were determined using two sample *t*-test, two-tailed distribution, (*) is *p* ≤ 0.05.

Compounds **1a-zd** were tested to determine their antiamoebic activity against *A. castellanii*. Results from amoebicidal assays reveal that compounds and their analogues showed effective antiamoebic activity against *A. castellanii* genotype T4 (*p* ≤ 0.05, two-tailed distribution) (Figure 1a,b). The structures of the tested compounds as well as their inhibitory effects are depicted in Supplementary Table S1. Among all the tested molecules, compounds **1m** and **1zb** showed the highest amoebicidal effects with 70% and 67% antiamoebic activity against *A. castellanii*, respectively (Figure 1a,b). Similarly, compounds **1zd** and **1i** showed 58% and 55% amoebicidal effects, respectively. Several compounds for instance, compounds **1d-j**, **1l**, **1n**, **1p-s**, **1v-x** and **1z** exhibited 40% to 50% amoebicidal activity (Figure 1a). Whereas, compounds **1a-c**, **1k**, **1o**, **1t**, **1u**, **1y**, **1za** and **1zc** did not show promising antiamoebic activity against *A. castellanii*. The potencies in term of 50% inhibitory effects of the most active compounds are summarized in Table 1. The results from IC_50_ indicated that the synthesized compounds exhibited 50% amoebicidal activities at micro molar concentration.

### 3.2. Compounds Prevented Amoebae Binding to Human Cells

**Figure 2:**
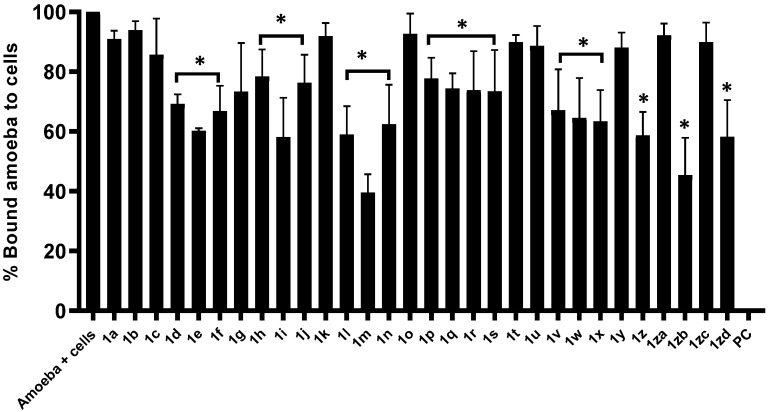
Compounds’ ability to block the *A. castellanii* binding to HeLa cells. Adhesion assays were accomplished to see whether *A. castellanii* interact with human cells. Note that compounds and their analogues suppressed *A. castellanii* significantly. *p* values were determined using two sample *t*-test, two-tailed distribution, (*) is *p* < 0.05. The data are presented as the mean ± standard error.

Compounds were evaluated for their competence to block amoebae-binding to human cells using adhesion assays. The overall results have shown that all the compounds with amoebicidal activity potentially blocked amoebae binding to HeLa cells (*p* < 0.05) (Figure 2). Similar to amoebicidal effects, compounds **1m** and **1zb** presented the highest activity (i.e., 61% and 55%, respectively) inhibition of amoebae binding to human cells (Figure 2). Compounds **1i**, **1l**, **1z** and **1zd** inhibited 42% amoebae binding to human cells. Similarly, compound **1e** showed 40% blocking ability to host cells (Figure 2). Compound with no amoebicidal activities failed to block amoeba binding to human cells. 

### 3.3. Compounds Noticeably Inhibited Amoebae Encystation and Excystation

**Figure 3:**
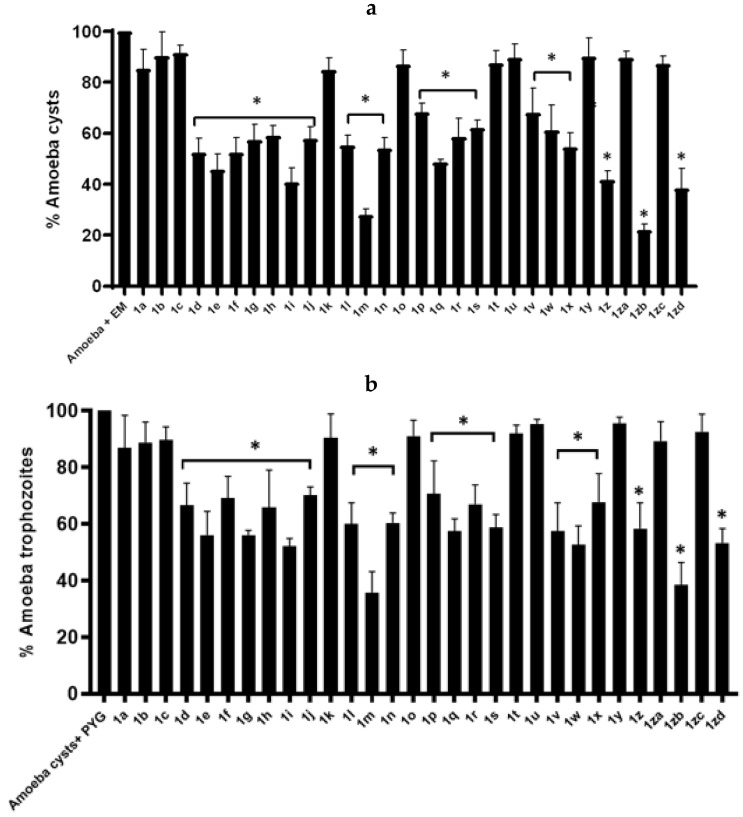
Compounds significantly inhibited both the encystation and excystation in *A. castellanii*. The results revealed that compounds and their analogues repressed the encystment as well excystment process when compared to the negative control. (**a**) Represents the encystation process while (**b**) Denotes the excystation effects. The data is presented as the mean ± standard error. *p* values were calculated using a two-sample *t*-test with a two-tailed distribution and (*) denotes that *p* ≤ 0.05.

Results from encystment and excystment assays revealed that the compounds significantly blocked both the encystation and excystation process in *A. castellanii* when compared to the negative control (*p* < 0.05) (Figure 3a,b). In encystation assays, compounds **1zb** and **1m** incredibly prevented trophozoites to cysts stage alteration in *A. castellanii*. Compound **1zb** and **1m** arrested 78% and 72% amoebae to convert into hardy cysts stage compared to the negative control (i.e., 100%) (Figure 3a). Likewise, compound **1zd** blocked 62% and compounds **1i** and **1z** prevented 60% encystation in *A. castellanii* (Figure 3a). All other compounds tested portray similar effects to amoebicidal assays against amoeba. For the excystation assays, *A. castellanii* cysts were significantly blocked from re-emerging as viable trophozoites, showing a similar pattern of action as encystation. (*p* < 0.05) (Figure 3b).

### 3.4. Compounds Offered Negligible Cytotoxic Properties against Human Cell Lines and Reduced Amoebae–Mediated Host Cell Cytotoxicity

**Figure 4:**
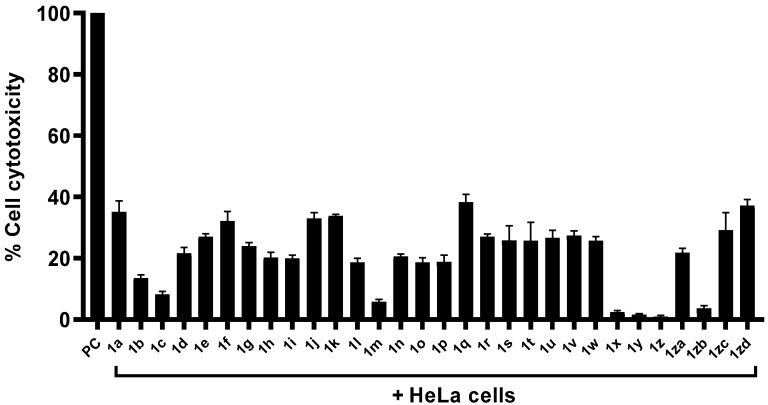
The impact of compounds and their analogues on human cells. Note that compounds and their analogues indicated marginal cytotoxic effects against human cell lines.

**Figure 5:**
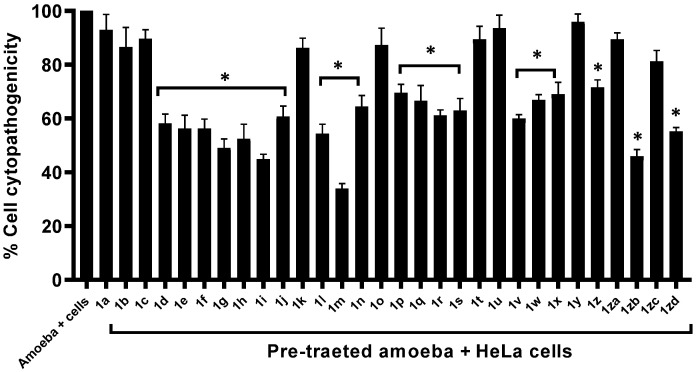
Pre-treatment of *A. castellanii* with compounds and their analogues remarkably decreased amoeba-mediated host cell death. Data is presented as mean ± standard error. *p* values were calculated using a two-sample *t*-test with a two-tailed distribution and (*) denotes that *p* ≤ 0.05.

To determine the cytopathic effects of compounds against human cells, Lactate dehydrogenase assays were conducted. Cytotoxicity results showed that the compounds showed minimal cytotoxicity against human cell lines (Figure 4). Compounds **1a**, **1q** and **1zd** revealed weak cytotoxic effects showing 35%, 38% and 37% cytotoxicity against HeLa cells, respectively. Results from MTT assays revealed that the compounds with most significant anti-amoebic activity presented limited cytotoxicity or higher cell viability against human cell lines. The values for EC_50_ and EC_90_ in Table 2 emphasize these compounds as a safe antiamoebic drugs used for its therapeutic usage. In some experiments, *A. castellanii* was pre-treated with all the compounds before being introduced into human cells. Cytopathogenicity results showed that compounds with eminent amoebicidal effects noticeably reduced amoebae-mediated host cell death when compared to negative control (i.e., amoeba + cells 100%) (Figure 5). Compound **1m** showed the highest activity reduced amoeba-mediated host death up to 33%. Similarly, compounds **1i** and **1zb** reduced the host cell death up to 45% and 46%, respectively (Figure 5).

## 4. Discussion

Free-living amoebae are ubiquitous in nature and thrive in diverse environmental niches including water, air and soil [6]. Given the opportunity, amoebae can cause a sight-threatening infection of the eye (AK) and a devastating infection of the CNS [2,8]. AK has two stages in its pathophysiology. There is better prognosis in the initial phase, as it is limited to the corneal epithelium. However, in the second phase, amoebae parasites infiltrate the underlying stroma, causing substantial collagen matrix degradation and causing severe inflammation [28]. Amoebae exist in two forms, namely the trophozoite and a hardy and resistant cyst form. These cysts are immune to most biocidal drugs and can endure harsh conditions, lingering in stromal tissues throughout treatment and excysting when the conditions become favorable, resulting in the reoccurrence of a vigorous infection [29]. Thus, the most difficult aspect of treating AK is eradicating the cysts, which are resistant to most antimicrobial medications [6,10,30]. Therefore, understanding the factors that cause *Acanthamoeba* encystation and identifying compounds which can eradicate the cysts or prevent encystation are of particular value. In the present study, the anti-parasitic efficacy of imidazothiazole derivatives compounds was assessed versus *A. castellanii*, in order to identify much needed compounds against these amoebae.

The amoebicidal assays results revealed that most of the compounds tested exhibited potent antiamoebic activity. Furthermore, the compounds were able to block amoebae encystation and excystation and prevented amoebae binding to human cells. Compounds showed negligible cytotoxic effects against human cells and pre-treatment of amoebae significantly reduced amoebae-mediated host cell cytotoxicity. These compounds were previously tested against cancer cells and most of them exerted promising activities [13,14,15,16,17]. Their molecular mechanism of action was shown to be via the inhibition of RAF kinases. It is thought that different proteases such as serine, metalloproteases and cysteine proteases are primarily involved in the encystation of *A. castellanii* [31]. The inhibition of these proteases could lead to the arresting of *A. castellanii* from trophozoites to cysts form. Moreover, *A. castellanii* treated with siRNA molecules considerably altered the growth rate and cellular viability [8]. However, the mechanism of action in the present study is not yet determined and is the subject of future studies. 

Of note, the compounds **1m** and **1zb** exhibited the most promising antiamoebic activity, whereas in the previous report, compound **1m** showed modest inhibitory effects against V600E-B-RAF and C-RAF kinases, while compound **1zb** was more potent. The IC_50_ values against V600E-B-RAF and C-RAF kinases were 15.8 and 158.0 nM, respectively [16]. Moreover, from all the compounds tested, compogunds **1m** and **1zb** showed 70% and 67% amoebicidal activity against amoebae trophozoites. This is in agreement with previous studies which have revealed that imidazothiazole derivatives are effective in treating several diseases and are indicative of these set of compounds as being promising drug leads [32]. Furthermore, the anti-bacterial, anti-viral and anti-fungal activity of imidazothiazole compounds has been reported in the literature, with examples of compounds such as ribavirin and fluconazole showing potent effects [32]. In a recent study it was reported that isavuconazonium sulfate prevented excystment in *A. castellanii* at 136 µM whereas the compounds tested in the present study arrested encystation and excystation at 50 µM [1]. This suggests that the compounds tested in the present study may have more impact on the growth and viability of *A. castellanii*. Importantly, the compounds showed minimal cytotoxic effects against human cell lines, which is encouraging for future development of these compounds for the treatment of AK infection. 

Upon considering the structure-activity relationship, it may be generally concluded that an amide linker is slightly more favorable than an amide spacer. Among the eleven most active derivatives (**1i**, **1m**, **1n**, **1q**, **1r**, **1v**, **1w**, **1x**, **1z**, **1zb** and **1zd**), six of them included an amide moiety. In addition, methoxy or hydroxyl group on the phenyl ring that is directly attached to the imidazothiazole nucleus was seen to be more optimal for activity than a fluoro group. Among the eleven most active compounds, there were six hydroxyl and four methoxy derivatives. The differences in activity encountered with the different derivatives might be affected by differences in the compound’s ability to penetrate the microbial cell wall and/or differences in affinity to the molecular target. In conclusion, the series of imidazothiazole derivatives in this study revealed significant antiamoebic activity with minimal cytotoxic effects. However, further research is needed to find out the precise mechanism(s) of action of these antiamoebic effects. Our results indicate that compounds **1m** and **1zb** in particular may be potential antiamoebic molecules, and should be considered in the future development of effective antiamoebic remedies. 

Key points

- Synthetic imidazothiazole derivatives showed novel antiamoebic properties
- Imidazothiazoles exhibited effects against both trophozoites and cysts
- Imidazothiazoles also interfere with encystation and excystation of *Acanthamoeba*

## Figures and Tables

**Table 1:** Compounds presented 50% inhibitory concentration (IC_50_) at µM concentrations.

Compounds No.	IC_50_ (µM)
**1i**	43.10
**1l**	59.90
**1m**	30.01
**1n**	47.49
**1q**	47.72
**1r**	64.69
**1v**	59.99
**1w**	46.29
**1x**	54.04
**1z**	44.80
**1zb**	32.42
**1zd**	41.01

**Table 2:** EC_50_ and EC_90_ of the most active compounds.

Compound No.	EC_50_ (µM)	MNTD/EC_90_ (µM)
**1i**	1411.0	90.94
**1m**	312.6	29.48
**1n**	648.1	16.45
**1q**	584.1	30.58
**1r**	183.2	33.89
**1v**	222.1	5.36
**1w**	304.2	36.06
**1x**	1274.0	102.10
**1z**	412.1	80.94
**1zb**	434.1	24.05
**1zd**	830.8	144.70

EC50 is Half maximal effective concentration; MNTD is Maximum Non-Toxic Dose.

## Data Availability

The datasets generated during and/or analyzed during the current study are available from the corresponding author on reasonable request.

## Supplementary Materials

The following supporting information can be downloaded at: https://www.mdpi.com/article/10.3390/antibiotics11091183/s1, Table S1. Structures of the tested target compounds and their amoebicidal activity at 50 µM concentration.
