# Supplementary material for: Antiamoebic Activity of Imidazothiazole Derivatives against Opportunistic Pathogen Acanthamoeba castellanii
Source: Antibiotics (Basel). 2022 Aug 31;11(9):1183. doi: 10.3390/antibiotics11091183 (PMC9494960; doi:10.3390/antibiotics11091183)
Supplement: Supplementary file 1 [file antibiotics-11-01183-s001.zip › antibiotics-1862839-supplementary.pdf]

Supplementary Table S1. Structures of the tested target compounds and their amoebicidal activity at 50 μM concentration.

Compound No.	Structure	Amoebicidal Activity Inhibition % ± SEM
**1a**	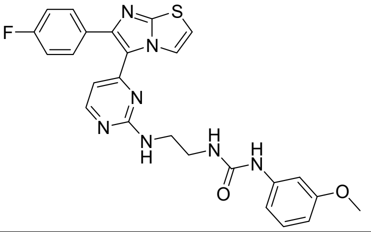	7.70% ± 3.57%
**1b**	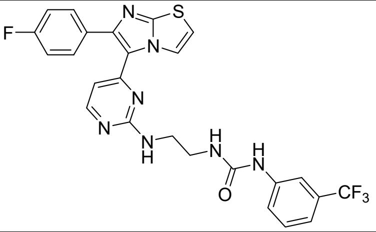	12.86% ± 5.93%
**1c**	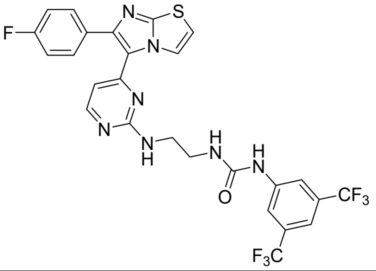	17.28% ± 1.88%
**1d**	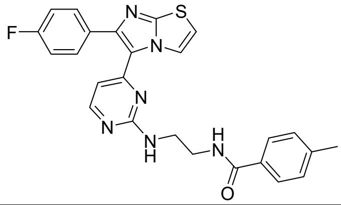	39.50% ± 1.53%
**1e**	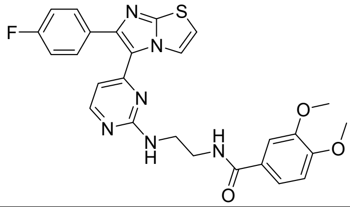	44.50% ± 1.79%
**1f**	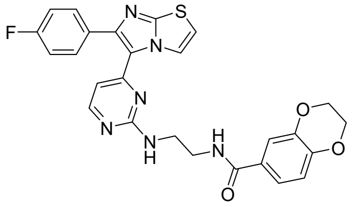	40.50% ± 4.84%

**1g**	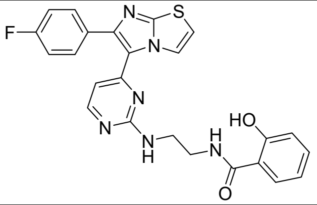	42.20% ± 3.29%
**1h**	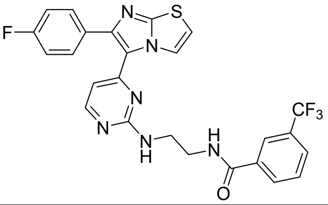	38.16% ± 3.74%
**1i**	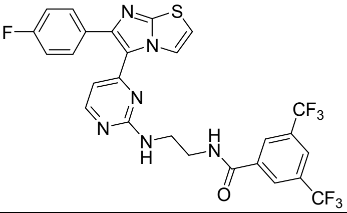	55.05% ± 1.25%
**1j**	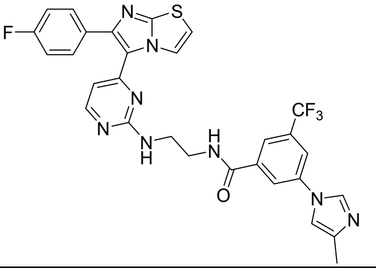	42.34% ± 3.81%
**1k**	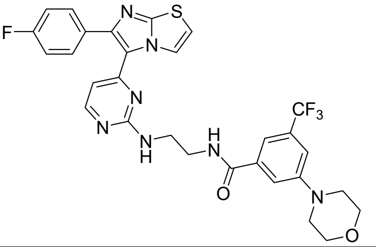	16.58% ± 6.08%
**1l**	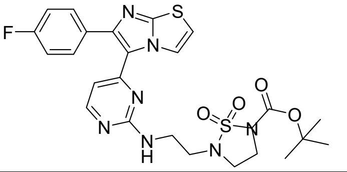	47.41% ± 1.67%
**1m**	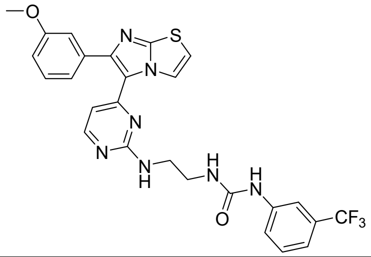	69.17% ± 2.39%

**1n**	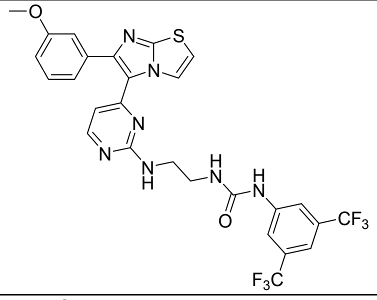	51.05% ± 2.36%
**1o**	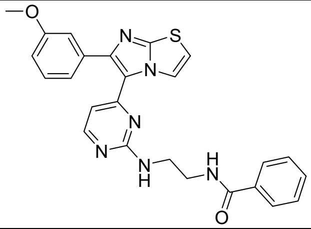	11.78% ± 11.34%
**1p**	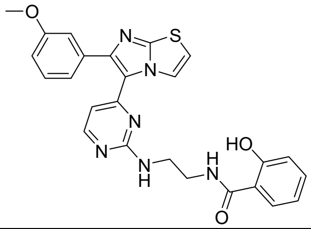	36.42% ± 5.43%
**1q**	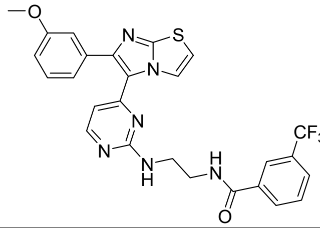	52.41% ± 5.31%
**1r**	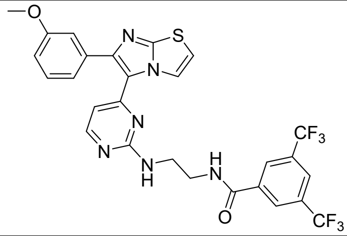	44.91% ± 8.42%
**1s**	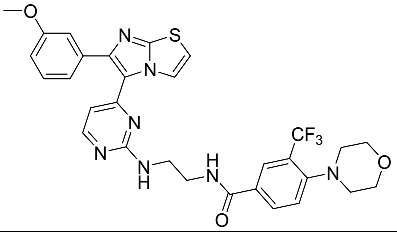	39.10% ± 2.31%

**1t**	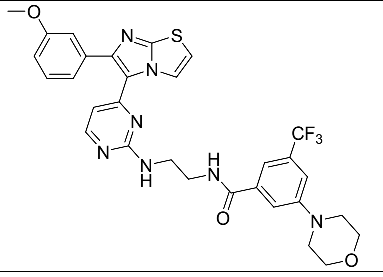	17.46% ± 3.75%
**1u**	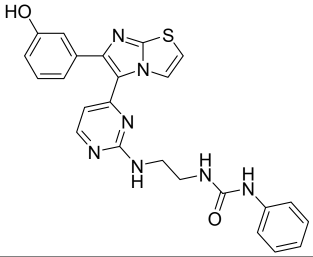	12.66% ± 5.04%
**1v**	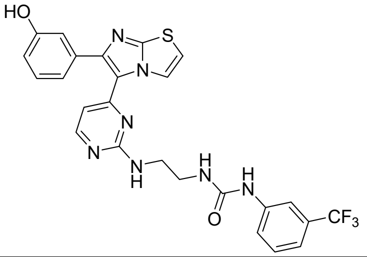	44.86% ± 2.98%
**1w**	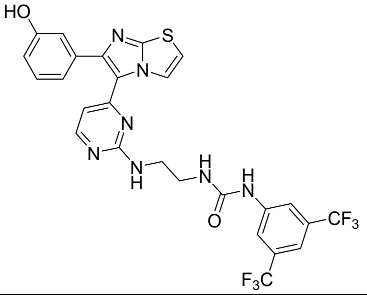	50.25% ± 4.67%
**1x**	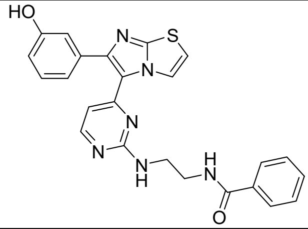	48.02% ± 3.69%
**1y**	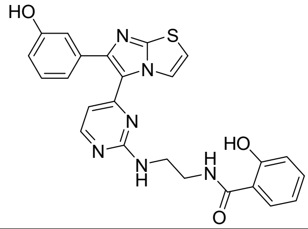	12.50% ± 4.68%

**1z**	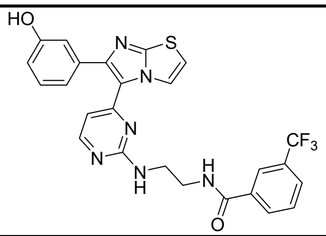	52.53% ± 1.67%
**1za**	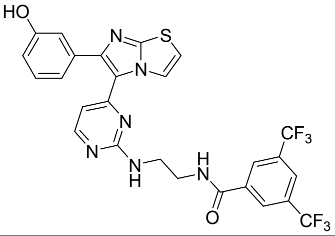	15.71% ± 6.61%
**1zb**	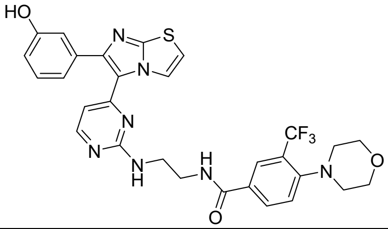	67.00% ± 3.19%
**1zc**	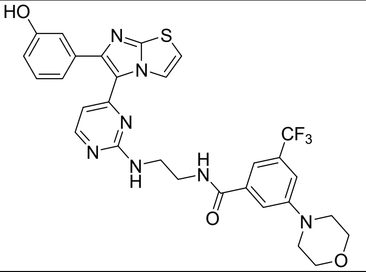	14.79% ± 3.19%
**1zd**	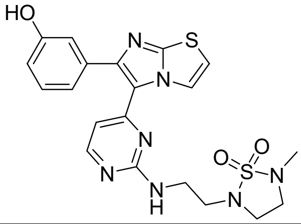	58.00% ± 3.41%
